# IGFBP-5 Promotes Fibrosis Independently of Its Translocation to the Nucleus and Its Interaction with Nucleolin and IGF

**DOI:** 10.1371/journal.pone.0130546

**Published:** 2015-06-23

**Authors:** Yunyun Su, Tetsuya Nishimoto, Carol Feghali-Bostwick

**Affiliations:** Department of Medicine, Division of Rheumatology & Immunology, Medical University of South Carolina, Charleston, South Carolina, United States of America; University of Texas Health Science Center at Houston, UNITED STATES

## Abstract

**Background:**

Insulin-like growth factor binding protein (IGFBP)-5 levels are increased in systemic sclerosis (SSc) skin and lung. We previously reported that IGFBP-5 is a pro-fibrotic factor that induces extracellular matrix (ECM) production and deposition. Since IGFBP-5 contains a nuclear localization signal (NLS) that facilitates its nuclear translocation, we sought to examine the role of nuclear translocation on the fibrotic activity of IGFBP-5 and identify IGFBP-5 binding partners relevant for its nuclear compartmentalization.

**Methods:**

We generated functional wild type IGFBP-5 and IGFBP-5 with a mutated NLS or a mutated IGF binding site. Abrogation of nuclear translocation in the NLS mutant was confirmed using immunofluorescence and immunoblotting of nuclear and cytoplasmic cellular extracts. Abrogation of IGF binding was confirmed using western ligand blot. The fibrotic activity of wild type and mutant IGFBP-5 was examined *in vitro* in primary human fibroblasts and *ex vivo* in human skin. We identified IGFBP-5 binding partners using immunoprecipitation and mass spectrometry. We examined the effect of nucleolin on IGFBP-5 localization and function via sequence-specific silencing in primary human fibroblasts.

**Results:**

Our results show that IGFBP-5-induced ECM production *in vitro* in primary human fibroblasts is independent of its nuclear translocation. The NLS-mutant also induced fibrosis *ex vivo* in human skin, thus confirming and extending the *in vitro* findings. Similar findings were obtained with the IGF-binding mutant. Nucleolin, a nucleolar protein that can serve as a nuclear receptor, was identified as an IGFBP-5 binding partner. Silencing nucleolin reduced IGFBP-5 translocation to the nucleus but did not block the ability of IGFBP-5 to induce ECM production and a fibrotic phenotype.

**Conclusions:**

IGFBP-5 transport to the nucleus requires an intact NLS and nucleolin. However, nuclear translocation is not necessary for IGFBP-5 fibrotic activity; neither is IGF binding. Our data provide further insights into the role of cellular compartmentalization in IGFBP-5-induced fibrosis.

## Introduction

Insulin-like growth factor binding protein (IGFBP-5) is one of six IGFBP proteins with high affinity binding to IGFs. IGFBPs serve as carriers for IGF and regulate IGF activity. IGFBPs also play IGF-independent functions. The effects of IGFBPs can be cell and tissue-specific. We previously reported that IGFBP-5 levels were increased in two fibrotic disorders, systemic sclerosis (SSc) and idiopathic pulmonary fibrosis (IPF) [1, 2]. Furthermore, IGFBP-5 exerted pro-fibrotic effects in primary fibroblasts [2]. In human skin maintained in organ culture, IGFBP-5 increased collagen bundle and dermal thickness [3]. In addition to its *in vitro* and *ex vivo* effects, IGFBP-5 induced fibrosis *in vivo* in mouse lung and skin [4–6].

The C-terminal domain of IGFBP-5 contains a putative nuclear localization signal (NLS) [7, 8]. This NLS is believed to mediate the translocation of IGFBP-5 to the nuclear compartment as IGFBP-5 was detected in the nucleus of the breast cancer cell line T47D, lung fibroblasts from patients with IPF, vascular smooth muscle cells and osteosarcoma cells [2, 7, 9–11]. The subcellular compartmentalization of IGFBP-5 can affect its function. For example expression of IGFBP-5 with a mutated NLS mainly localized to the cytoplasm and showed enhanced proliferation and migration compared to wild type IGFBP-5. Wild-type IGFBP5 translocated to the nucleus where it exerted inhibitory effects on proliferation and migration of MDA-MB-435 cells suggesting that IGFBP-5 cellular compartmentalization dictates its role in breast cancer cell metastasis [12]. In primary fibroblasts, IGFBP-5 induced Egr-1 and its nuclear localization [9]. In addition, trafficking of IGFBP-5 was dependent on caveolin-1 as loss of caveolin-1 promoted the accumulation of IGFBP-5 in the extracellular milieu [13]. Since we and others have reported nuclear localization of IGFBP-5 [2, 7, 9–11] which parallels its activation of transcription factors such as Egr-1, we sought to examine the role of nuclear translocation on the fibrotic activity of IGFBP-5.

Identification of IGFBP-5 binding partners can provide insights into the mechanisms mediating IGFBP-5 effects. Some of the IGFBP-5 binding partners have been identified. For example, IGFBP-5 was shown to interact with four and a half LIM domains protein (FHL)-2 in osteoblast-like cells [10] to coordinate bone formation. IGFBP-5 also was shown to interact with and serve as a substrate for pregnancy-associated plasma protein-A2 (PAPPA2) [14, 15], a specific protease that cleaves circulating IGFBP-5. IGFBP-5 interacted with Ras-association domain family 1 protein (RASSF1C) [16] to regulate osteoblast cell proliferation.

Although IGFBP-5 was originally identified as an IGF-binding protein, increasing numbers of IGF-independent functions have been identified [11, 17–21]. For example in myogenesis, IGFBP-5 exerted IGF-dependent effects on myoblasts, but its effects on cell survival and its anti-apoptotic functions were IGF-independent [20]. IGFBP-5 induced breast cancer cell MCF-7 adhesion and inhibited its migration in an IGF-independent manner [21]. In addition, the transactivation activity of IGFBP-5 in vascular smooth muscle cells was reported to occur independently of IGF [11].

Our goal was to determine whether the NLS is required for IGFBP-5 to exert pro-fibrotic effects and whether its IGF binding capacity was necessary for its induction of ECM. We also sought to identify IGFBP-5 binding partners in primary human fibroblasts.

## Materials and Methods

### Adenovirus Constructs

The full length cDNA of human IGFBP-5 was generated as previously described [2]. The cDNA was subcloned into the shuttle vector pAdlox with a C-terminal triplicate (3x) Flag tag and used for the generation of replication deficient adenovirus expressing IGFBP-5-Flag. Mutant constructs were generated by GeneWiz (South Plainfield, NJ). For the NLS mutant, amino acid residues 214–218 consisting of RGRKR were mutated to MDGEA [8]. For the IGF binding mutant, amino acid residues 68–74 were mutated from KPLHALL to NQQHAQQ [22]. Mutant IGFBP-5 constructs were also used for the generation of replication-deficient adenoviruses in the Vector Core facility of the University of Pittsburgh.

### Primary human lung fibroblast culture

Primary human lung fibroblasts were cultured from normal donor lungs following written consent as previously described [2] under a protocol approved by the University of Pittsburgh Institutional Review Board (IRB). Cells were used in passages 4 to 7. Fibroblasts were infected with adenoviruses at a multiplicity of infection (MOI) of 50. Normal human skin remnants from plastic surgery were obtained following written consent under a protocol approved by the University of Pittsburgh IRB.

### Small interfering RNA (siRNA) transfection

Primary human lung fibroblasts were seeded in six-well plates 24–48 hours prior to transfection with siRNA. Nucleolin sequence-specific siRNA and negative control scrambled siRNA were purchased from Applied Biosystems/Ambion (Austin, TX). Transfection was done using Lipofectamine 2000 (Invitrogen, Grand Island, NY) and 100pmol siRNA diluted in Opti-MEM I Reduced-Serum Medium (Life Technologies, Grand Island, NY) following the manufacturer’s recommendation.

### Fibroblast nuclear and cytoplasmic extracts

Nuclear and cytoplasmic extracts were prepared as previously described [23] with some modifications. Briefly, 5–6 x 10^6^ fibroblasts were pelleted and resuspended in 200 μl of Buffer A [10 mM HEPES-KOH pH 7.9, 1.5 mM MgCl_2_, 10 mM KCl, 0.5 mM dithiothreitol, supplemented with protease inhibitors cocktail (Sigma, St Louis, MO)] and incubated on ice for 10 minutes. After centrifugation at 12,000 rpm for 30 seconds, supernatant (cytoplasmic extract) was harvested [23]. The pellet was washed three times using 1 ml buffer A supplemented with 0.2%NP-40 to remove any residual cytoplasmic extracts and peri-nuclear proteins. The pellet was then resuspended in 50 μl Buffer C (20 mM HEPES-KOH pH7.9, 25% glycerol, 420 mM NaCl, 1.5 mM MgCl_2_, and 0.2 mM EDTA) and protease inhibitors cocktail and incubated on ice for 20 minutes with intermittent vortexing. After centrifugation at 12,000 rpm for 5 minutes, the supernatant was collected and used as a source of nuclear proteins.

### Ex vivo human skin culture

Human skin was maintained in organ culture as described previously [3]. Adenoviruses (1 x 10^8^ pfu in 100 μl of 1xPBS) were injected intradermally. Skin was harvested after 10 days, fixed in 10% formalin, and embedded in paraffin. Sections were stained with hematoxylin and eosin (H&E). Images were captured on an Olympus Provis 2 microscope (Olympus Corporation, Melville, NY). Dermal thickness was measured using Image J. Thickness was measured in 4 random fields from each sample. The experiment was repeated using skin from four different donors.

### Western ligand blot

Western ligand blot to detect IGF binding activity was done as previously described [2]. Briefly, 25 μg of protein was resolved on SDS-PAGE under non-reducing conditions. Proteins were transferred to PVDF membrane. The membrane was blocked with 5% non-fat dry milk at room temperature for one hour, incubated with 0.5 μg/ml of biotinylated IGF-I (GroPep, Thebarton, SA, Australia) in TBS/Tween-20 (TBST) at 4°C overnight, washed with TBST, and incubated with Streptavidin-HRP (Amersham, Piscataway, NJ) for one hour at room temperature, then washed again. Signal was detected using Chemiluminescence (Perkin Elmer, Waltham, Massachusetts). Images were analyzed using Image J.

### Western blot

For immunoblotting (IB), whole cell lysates were prepared by scraping cells directly in 2x SDS sample buffer. Extracellular matrix was prepared as we previously described [2]. Proteins were separated by SDS-PAGE and transferred to membranes. Membranes were blocked with 5% non-fat dry milk in TBST buffer then incubated with antibodies against IGFBP-5 (Gropep, Thebarton, SA, Australia), fibronectin, collagen 1A1, GAPDH, tenascin-C, nucleolin (Santa Cruz, Dallas, TX), Histone H3 (Sigma, St. Louis, MO) or tubulin (Epitomics Inc, Burlingame, CA), washed with TBS three times, then incubated with horseradish peroxidase-labeled secondary antibody (Santa Cruz, Dallas, TX). Signals were detected by chemiluminescence (Perkin Elmer, Waltham, MA). Images were analyzed using Image J.

### Immunoprecipitation

Human lung fibroblasts were infected at a MOI of 50 with replication-deficient adenoviruses encoding IGFBP-5-3x Flag or control vector encoding 3x Flag for 72 hours. Cells were lysed in lysis buffer (50mM Tris HCl, pH 7.4, 150mM NaCl, 1mM EDTA, 1%Triton X-100) supplemented with protease inhibitors cocktail (Sigma, St Louis, MO). A total of 200 μg lysates was incubated with 40 μl of gel suspension EZview Red anti-Flag M2 affinity gel (Sigma, St Louis, MO) overnight at 4°C, beads were washed with 1ml TBS (50mM Tris HCl, 150mM NaCl, pH 7.4) three times, and the binding proteins were eluted by boiling in 30 μl of 2x SDS sample buffer. Proteins were resolved on 10%SDS-PAGE and gels were stained with Coomassie Blue. Gel bands were excised and proteins were identified by Mass Spectrometry in the Proteomics Core of the University of Pittsburgh. In some experiments, lysates were incubated with anti-nucleolin antibody-bound agarose beads for immunoprecipitation (IP), and subjected to immunoblotting.

### Immunofluorescence

Human lung fibroblasts were seeded in chamber slides (BD Biosciences, Bedford, MA) and infected with replication-deficient adenoviruses encoding IGFBP-5 or NLS-mutant IGFBP-5 for 72 hours. Cells were fixed with 2% of paraformaldehyde for 15 minutes, permeabilized with 0.1% TritonX-100 for 15 minutes, blocked with 5% of goat normal serum for one hour, and incubated with anti-IGFBP-5 antibody (Gropep, Thebarton, SA, Australia) overnight at 4°C. Slides were washed with 1X PBS three times, incubated with biotinylated secondary antibody for one hour at room temperature, followed by Texas Red Avidin D (Vector Labs, Burlingame, CA). Hoechst (Sigma, St Louis, MO) was used to identify nuclei. Images were taken on an Olympus Provis 3 microscope (Olympus Corporation, Melville, NY).

### Hydroxyproline assay

Skin collagen content was measured using hydroxyproline assay as previously described [24].

### Statistical analysis

All continuous variables were expressed as the mean ± standard deviation. Comparison among 3 or more groups was performed using One-way ANOVA followed by the student’s *t* test. The significance level was set at *P* <0.05.

## Results

### Generation of mutant IGFBP-5

**Fig 1:**
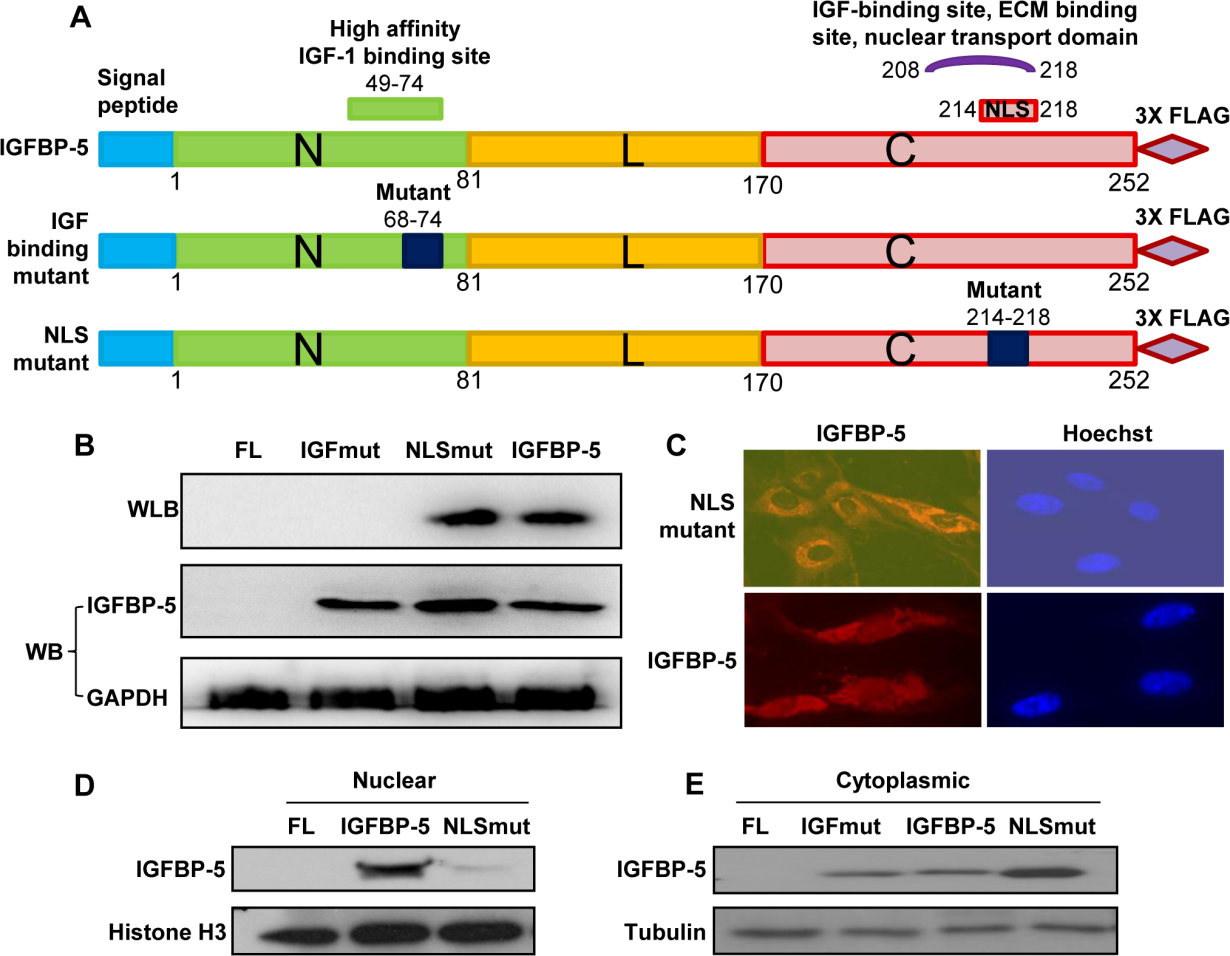
Generation of IGFBP-5 with mutated IGF binding and NLS domains and validation of the loss of function imparted by the mutations. (A) Schematic of wild type, IGF binding site-mutant, and NLS-mutant IGFBP-5 expressing constructs used for the generation of adenoviruses. (B) Confirmation that all three constructs result in IGFBP-5 production by western blotting and validation of the loss of binding to IGF of the IGF-binding site mutant using western ligand blotting. (C) The NLS-mutant IGFBP-5 does not translocate to the nucleus of primary human fibroblasts as detected using immunofluorescence. (D) The NLS-mutant IGFBP-5 is not detected in nuclear extracts of primary human fibroblasts expressing the indicated constructs, but its levels were higher in cytoplasmic fractions as detected following cellular fractionation and immunoblotting.

The domains of IGFBP-5 that bind to IGF-I localize to the N-terminal domain at aa49-74 and C-terminal domain at aa208-218, with the N-terminal domain being the high affinity binding site while the C-terminal region contains a low affinity binding site. This latter site overlaps with the ECM binding site and the NLS. To delineate the role of the NLS and IGF-binding domains in fibrosis, we generated three IGFBP-5 constructs: a wild type human IGFBP-5 [2] to which we added a FLAG tag, IGFBP-5 with a mutated NLS with aa214-aa218 mutated from RGRKR to MDGEA [8], and IGFBP-5 with a mutated IGF binding domain with aa68 to aa74 mutated from KPLHALL to NQQHAQQ [22] (Fig 1A).

Primary human fibroblasts expressing wild type, NLS mutant, and IGF mutant IGFBP-5 expressed and secreted comparable levels of IGFBP-5 as detected by western blot (Fig 1B). Western ligand blotting confirmed that the wild type and NLS mutant IGFBP-5 proteins maintained IGF binding whereas the binding of the IGF-mutant IGFBP-5 to IGF-I was abrogated.

To confirm that the NLS mutation blocked IGFBP-5 translocation to the nucleus, immunofluorescence was used to localize IGFBP-5 in fibroblasts expressing wild type and NLS mutant IGFBP-5. Wild-type IGFBP-5 was distributed in the cytoplasm and nucleus of the cells, but the NLS mutant was mainly perinuclear and cytoplasmic in distribution (Fig 1C). This was further confirmed by cellular fractionation of fibroblasts that showed IGFBP-5 was undetectable in cells expressing the NLS mutant (Fig 1D), whereas cytoplasmic levels of IGFBP-5 were higher compared to wild type IGFBP-5 (Fig 1E). These findings also confirm that an intact NLS is necessary for IGFBP-5 translocation to the nucleus in primary human fibroblasts.

### IGFBP-5 induces ECM production *in vitro* independently of its nuclear translocation and binding to IGF

**Fig 2:**
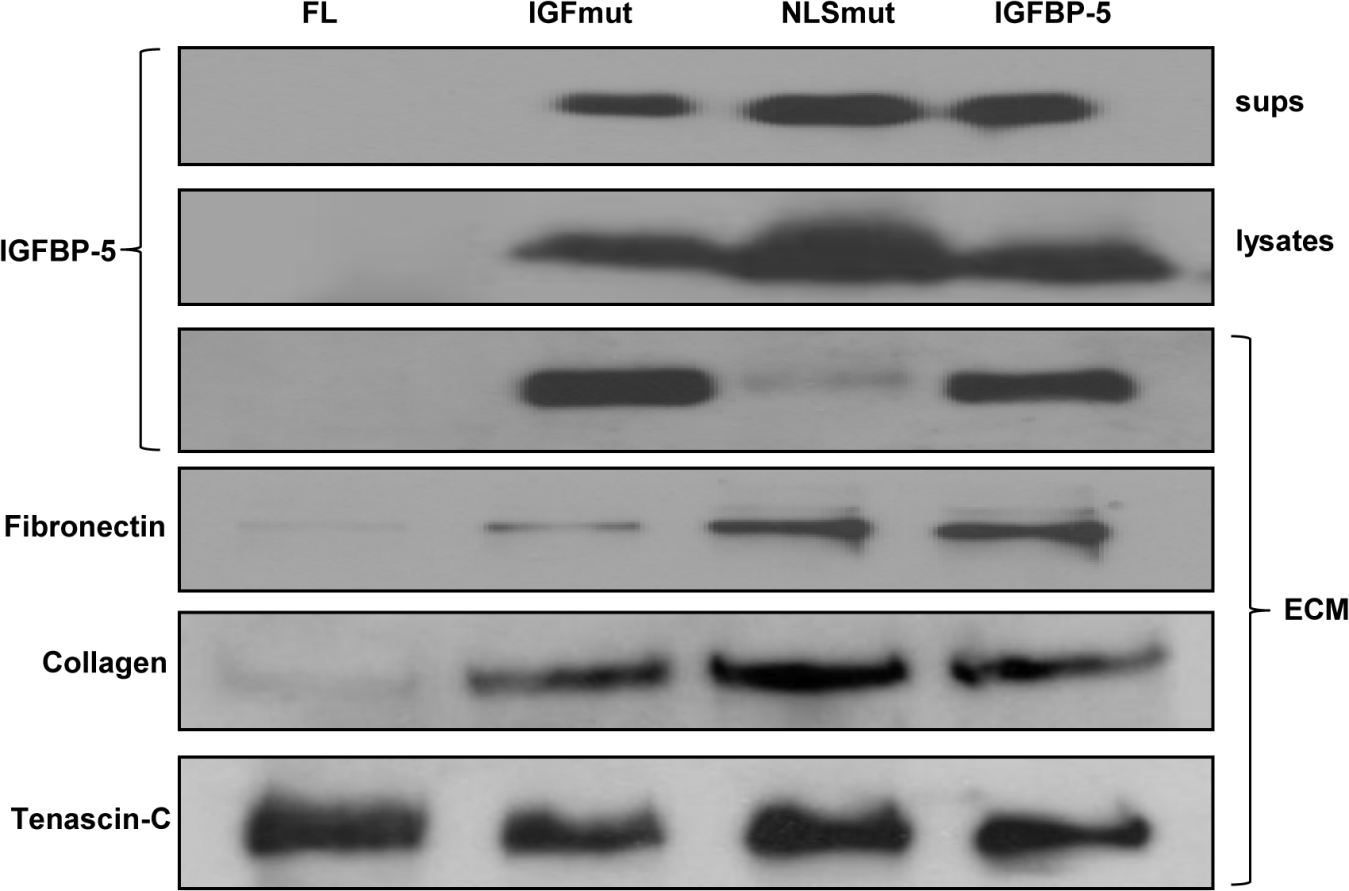
Mutant IGFBP-5 exerts *in vitro* pro-fibrotic activity that is similar to that of wild-type IGFBP-5. Primary human fibroblasts were infected with adenovirus expressing wild type IGFBP-5, IGF binding site-mutant IGFBP-5, or NLS-mutant IGFBP-5. Cell culture supernatants (sups), cellular (lysates), and extracellular (ECM) fractions were analyzed by immunoblotting.

We had previously reported that IGFBP-5 induces ECM production in primary human fibroblasts [2]. We now show that addition of a C-terminal Flag tag to IGFBP-5 does not hinder its ECM promoting effects (Fig 2). To determine if the pro-fibrotic effect of IGFBP-5 requires its translocation to the nucleus or its binding to IGF, we compared the effect of expressing wild-type IGFBP-5, the NLS mutant, and the IGF-binding mutant on ECM production. When compared to wild type IGFBP-5, expression of NLS mutant IGFBP-5 induced production of collagen and fibronectin to similar levels (Fig 2). The IGF-binding mutant had a more modest effect on ECM production but still increased collagen and fibronectin levels compared to the control. It is noteworthy that the NLS mutant showed very little deposition of IGFBP-5 in the ECM (Fig 2). This is likely due to the fact that the NLS and ECM binding domains overlap, and thus mutating the NLS domain abolished ECM binding. In spite of the fact that very low levels of NLS mutant IGFBP-5 were detected in the ECM, fibronectin and collagen levels were increased to levels comparable to those induced by wild type IGFBP-5 (Fig 2). These results imply that neither the NLS sequence nor the IGF-binding domain is necessary for the ECM-promoting effects of IGFBP-5.

### IGFBP-5 induces fibrosis *ex vivo* independently of its nuclear translocation and binding to IGF

**Fig 3:**
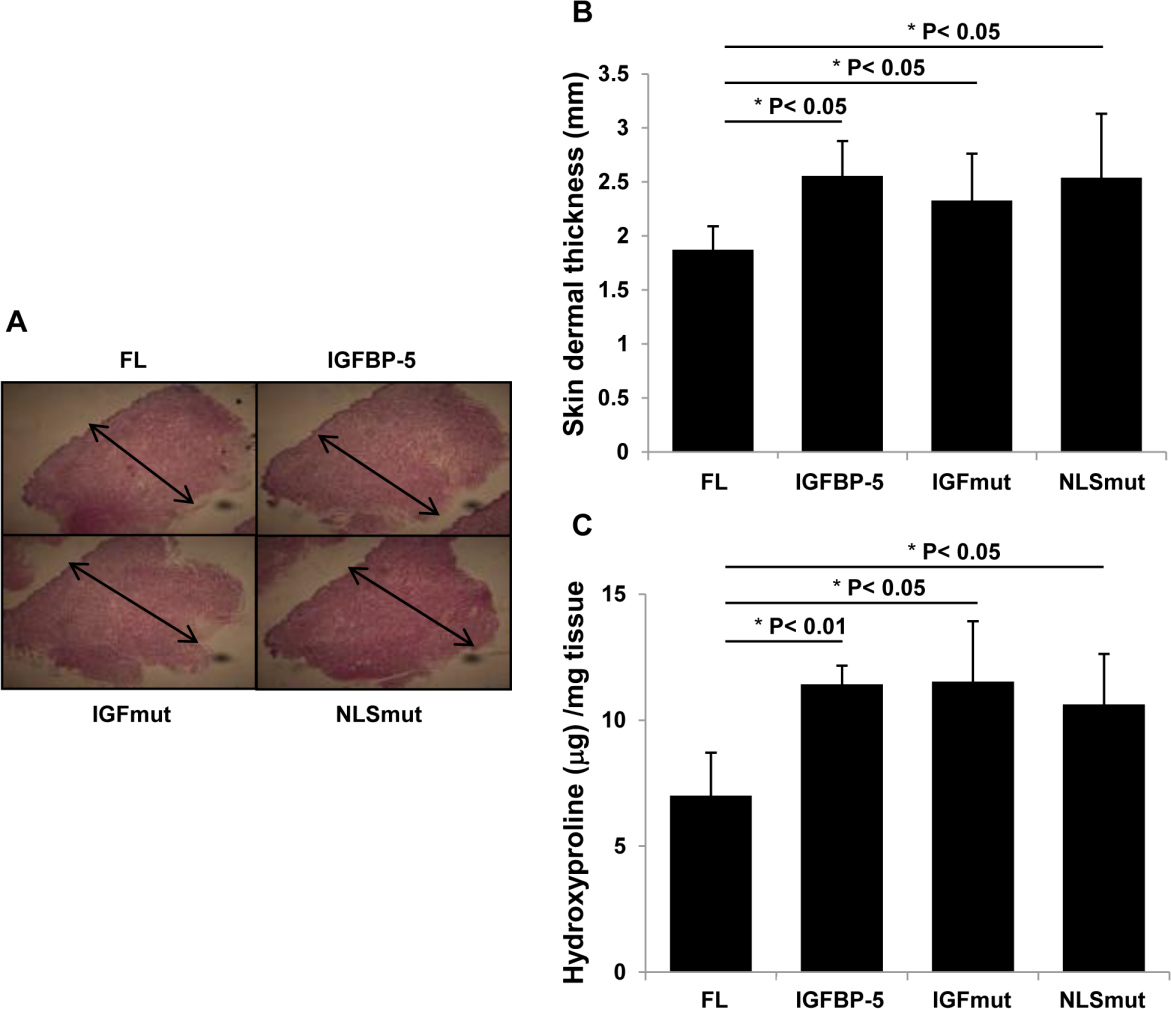
Mutant IGFBP-5 is pro-fibrotic *ex vivo* in human skin. Human skin from four different donors was injected with the indicated IGFBP-5 expressing adenoviruses and maintained in organ culture for 10 days. (A) Skin was harvested and examined histologically. (B) Dermal thickness was measured in human skin explants. (C) ECM was quantified using hydroxyproline assay. Data in (B) and (C) represent mean and standard deviation.

To extend the *in vitro* findings, we engineered human skin maintained in organ culture to express wild type IGFBP-5, the NLS mutant, and the IGF-binding mutant. All three proteins exerted similar pro-fibrotic effects in that they induced dermal thickness in human skin, as measured on H&E-stained sections of paraffin-embedded skin samples (Fig 3A and 3B). The increase in dermal thickness paralleled increased hydroxyproline levels in skin from four different donors (Fig 3C). The *ex vivo* results further confirm that mutating the NLS or IGF-binding domains of IGFBP-5 does not impact its pro-fibrotic effect.

### Nucleolin is an IGFBP-5 binding protein

**Fig 4:**
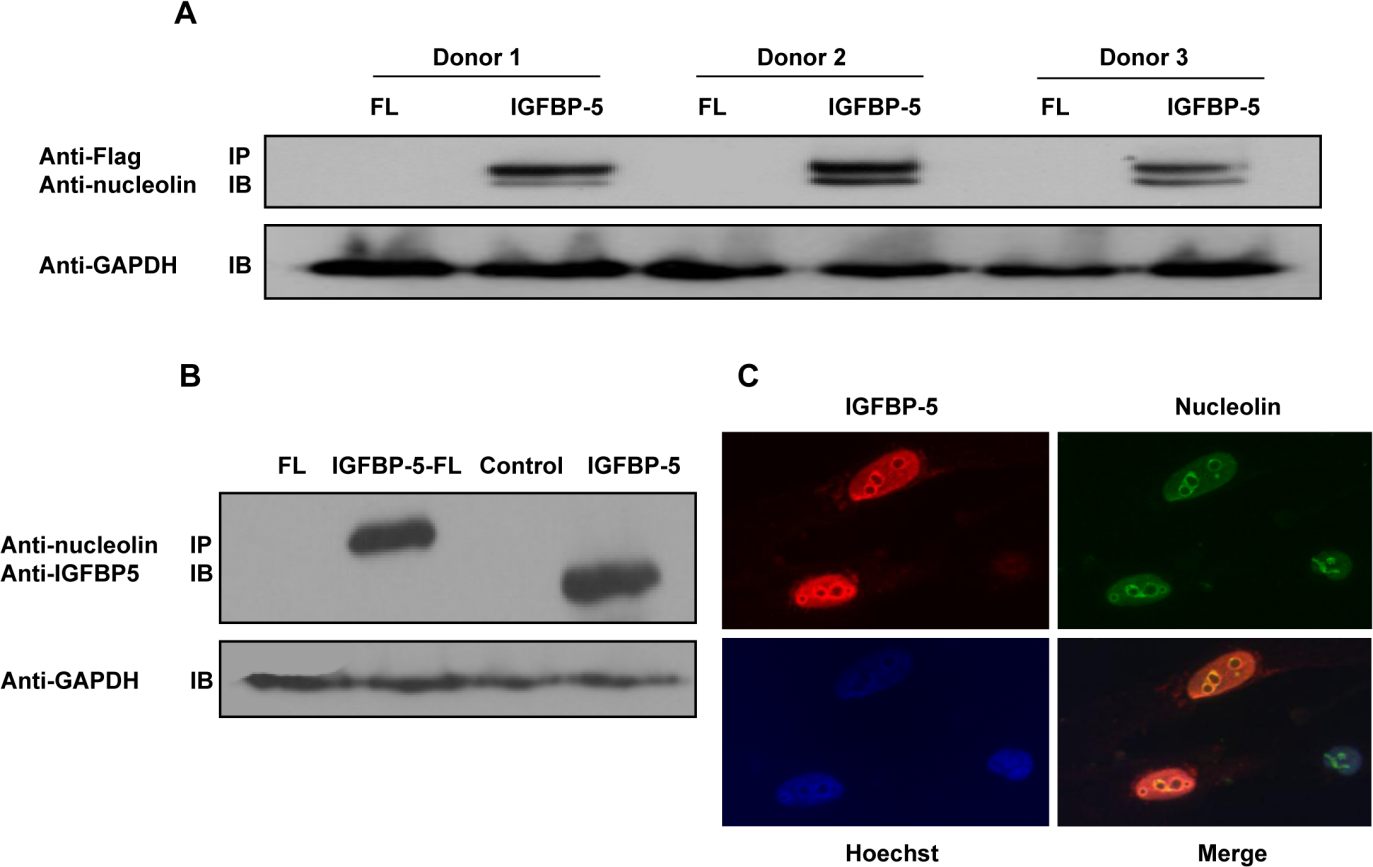
Nucleolin is an IGFBP-5 binding partner. Following identification of nucleolin using mass spectrometry, interaction with IGFBP-5 was confirmed using co-precipitation. (A) Wild type IGFBP-5 binds nucleolin in fibroblasts from three different donors. (B) Reverse pull-down with nucleolin antibody confirms the interaction with IGFBP-5. GAPDH in input lysate was detected to confirm that lysate fractions with equivalent protein levels were used for the IP. (C) Co-localization of nucleolin and IGFBP-5 is detected using immunofluorescence (IGFBP-5: red; nucleolin: green).

To identify proteins that may interact with IGFBP-5 in primary human fibroblasts and contribute to its pro-fibrotic activity, we immunoprecipitated IGFBP-5 and identified binding partners using mass spectrometry. Using three independent immunoprecipitation assays, we identified nucleolin as an IGFBP-5 binding protein. The interaction with nucleolin was confirmed using cellular lysates from fibroblasts derived from three different donors (Fig 4A). The IGFBP-5-nucleolin interaction was confirmed by reverse IP using anti-nucleolin antibody (Fig 4B). Furthermore, nucleolin bound both wild type IGFBP-5 and IGFBP-5 with a C-terminal Flag tag suggesting that the tag does not disrupt binding of IGFBP-5 to nucleolin (Fig 4B). Using immunofluorescence, we show that IGFBP-5 and nucleolin co-localize in the nucleus of primary human fibroblasts (Fig 4C).

**Fig 5:**
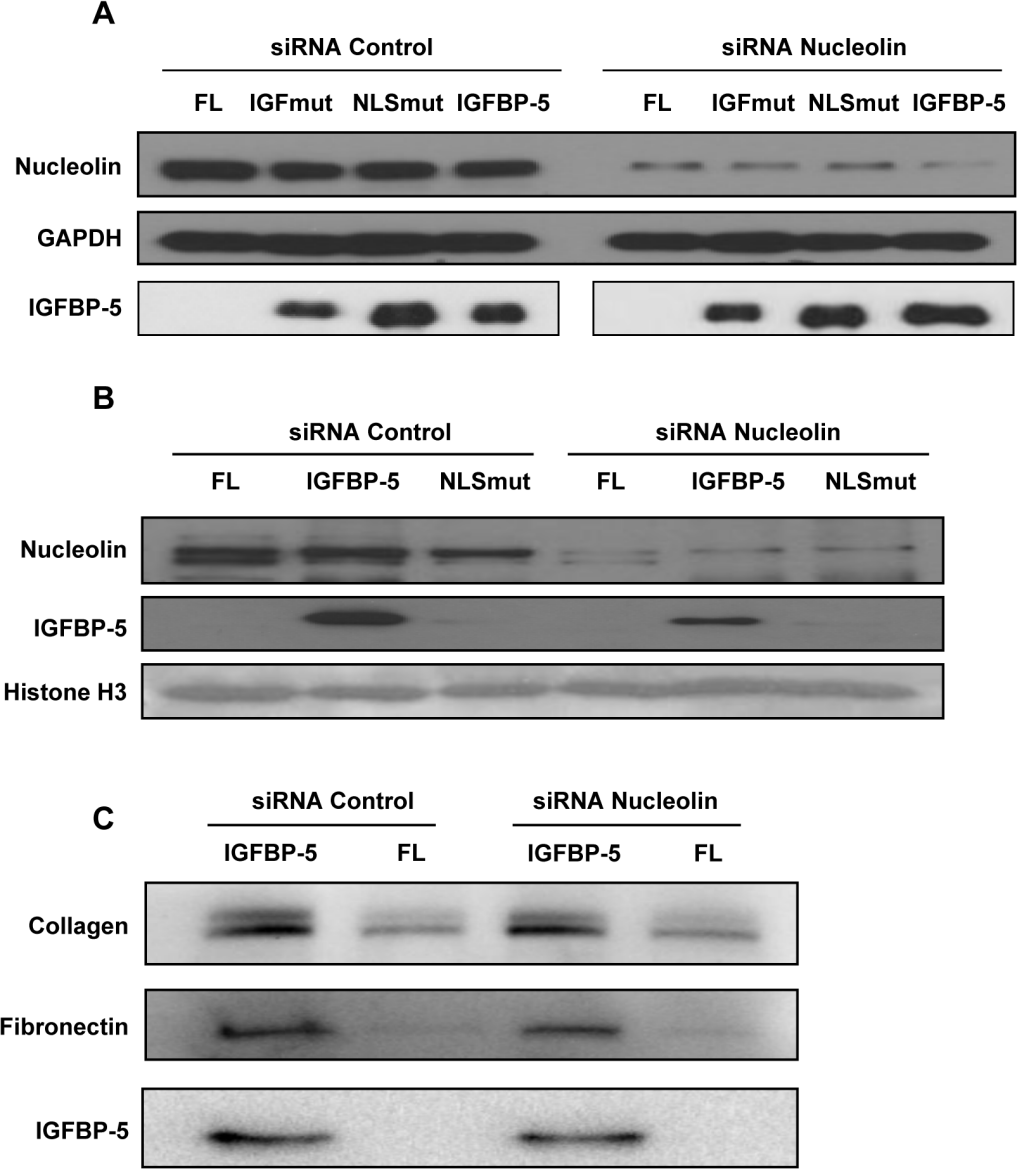
Silencing nucleolin does not alter the ECM-promoting effects of IGFBP-5. (A) Nucleolin is efficiently silenced using sequence-specific siRNA. Silencing nucleolin has no effect on IGFBP-5 production. (B) Silencing nucleolin inhibits translocation of IGFBP-5 to the nucleus as detected by immunoblotting of nuclear extracts. Histone H3 is used to confirm nuclear localization. (C) Silencing nucleolin does not alter the ECM-promoting effects of IGFBP-5 as detected using immunoblotting.

**Fig 6:**
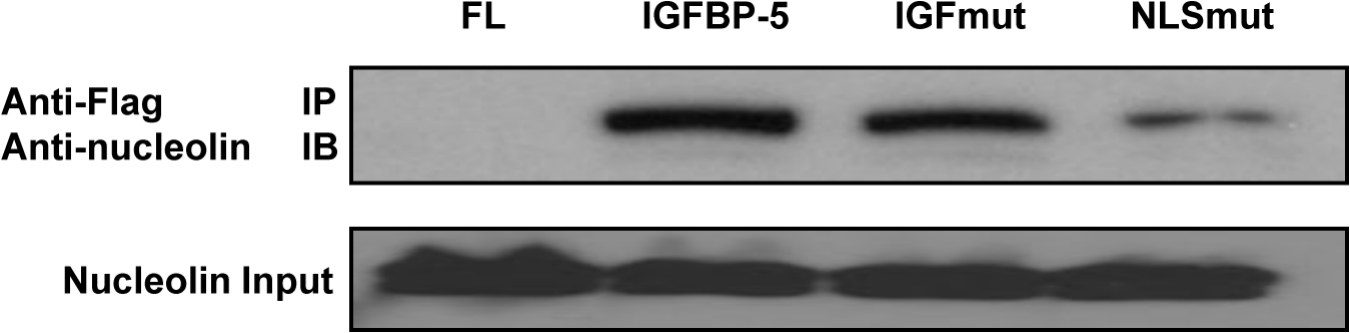
Mutating the NLS domain of IGFBP-5 reduces its ability to bind nucleolin. The interaction of Flag-tagged IGFBP-5 and nucleolin was detected using immunoprecipitation using anti-Flag antibody followed by immunoblotting to detect nucleolin. Input nucleolin was detected in lysates prior to co-precipitation to ensure that samples contained equivalent amounts of protein.

To determine if the pro-fibrotic effects of IGFBP-5 require nucleolin, we silenced nucleolin in primary human fibroblasts and expressed wild-type and mutant IGFBP-5. Efficient silencing of nucleolin using sequence-specific siRNA was observed as nucleolin protein levels following silencing decreased by 65% compared to the control siRNA (Fig 5A). Silencing nucleolin did not change levels of secreted IGFBP-5 (Fig 5A) but reduced IGFBP-5 translocation to the nucleus (Fig 5B). These findings suggest that IGFBP-5 translocation to the nucleus is dependent on nucleolin. However, silencing nucleolin did not block the ability of IGFBP-5 to induce ECM production (Fig 5C). Co-immunoprecipitation of wild type and NLS mutant IGFBP-5 showed decreased interaction of the NLS mutant with nucleolin (Fig 6). These findings suggest that IGFBP-5 may interact with nucleolin via the NLS domain, and that its interaction with nucleolin may be necessary for the translocation of IGFBP-5 to the nucleus. Since mutating the NLS of IGFBP-5 did not impact its ECM-inducing effects, it is not surprising that reducing IGFBP-5 translocation to the nucleus by silencing nucleolin also did not abrogate the ECM-promoting effects of IGFBP-5.

## Discussion

IGFBP-5 is known to exert both IGF-dependent and independent effects. Identification of IGFBP-5 binding partners would provide insights into the mechanisms mediating IGFBP-5 effects. IGFBP-5 has been shown to interact with and serve as a substrate for PAPPA2 [14, 15]. Further, IGFBP-5 nuclear localization in vascular smooth muscle cells resulted in transcriptional regulatory activity [11]. Our group showed that caveolin-1 interacted with IGFBP-5 and coordinated trafficking of IGFBP-5 from the plasma membrane to the nucleus [13]. To assess the functional importance of the IGF-binding and NLS domains for IGFBP-5’s pro-fibrotic effects, the effect of NLS-mutant and IGF-binding mutant IGFBP-5 was compared to that of wild-type IGFBP-5. All three IGFBP-5 constructs retained fibrotic activity, promoted fibronectin and collagen deposition in the ECM, and increased skin thickness to a similar degree. Our results demonstrate that IGFBP-5 translocation to the nucleus and its binding to IGF are not required for its fibrosis-promoting effects.

IGFBP-5 binds ECM components such as collagen, laminin, fibronectin thrombospondin-1, osteopontin and vitronectin [25–28], as part of its IGF modulating function and/or for protection of ECM components from proteolytic degradation [13, 27, 28]. We currently show that IGFBP-5 binds nucleolin. Nucleolin mainly localizes to the nucleolar compartment and was originally described to function in ribosome biogenesis [29]. More recently, novel roles for nucleolin in several cellular processes such as phosphorylation, glycosylation and methylation have been reported [30–33]. Nucleolin contains a nucleic acid binding domain and functions in the regulation of transcription, posttranscriptional processing, DNA metabolism, cell cycle, and cell proliferation [34–36]. Nucleolin is also described as a receptor for growth factor midkine (MK) [37], pleiotrophin (PTN) [38], and endostatin [39]. Nucleolin is able to translocate from the nucleus to the cell surface and serve as a receptor for *Francisella tularensis* [40]. Thus nucleolin is a pleiotropic protein with multiple functions. We now add a novel function to the repertoire of activities attributed to nucleolin: as nuclear receptor for IGFBP-5 that is responsible for the translocation of IGFBP-5 to the nucleus. Importin β was reported to mediate nuclear import of IGFBP-5 [8]. Our findings suggest that nucleolin is necessary for IGFBP-5 trafficking to the nucleus. However, since silencing nucleolin did not abrogate the ability of IGFBP-5 to induce ECM production, and since mutating the IGFBP-5 NLS did not abrogate the protein’s pro-fibrotic effects, we conclude that nucleolin and nuclear trafficking of IGFBP-5 are not required for its ECM-promoting activity. Our findings do not rule out the possibility that nucleolin may contribute to some of the other functions attributed to IGFBP-5 in other cell types, such as proliferation, migration, differentiation, or survival/apoptosis.

Our efforts have focused on delineating the mechanisms mediating the pro-fibrotic activity of IGFBP-5. To that end, we showed that DOK5/IRS6 is a downstream mediator of IGFBP-5 function [41] and that IGFBP-5 localizes to caveolae [13]. We also showed that IGFBP-5 induces Egr-1, and that Egr-1 is required for IGFBP-5 induction of ECM production [9]. Further, several of the effects of IGFBP-5 were dependent on MAPK activation [9, 41]. The current findings suggest that the pro-fibrotic activity of IGFBP-5 is independent of its nuclear translocation. This supports our previous findings showing reduced intracellular IGFBP-5 in cells deficient in caveolin-1 with maintenance of a fibrotic phenotype when caveolin-1 levels are reduced. This could be attributed, at least in part, to the ECM-promoting activity of extracellular IGFBP-5 [13]. Other effects of IGFBP-5 have been the focus of research in the cancer field. IGFBP-5 was shown to mediate proliferation and cell migration [21]. Thus, the nuclear localization of IGFBP-5 may facilitate the protein’s modulation of cell proliferation, migration, differentiation, and survival but not necessarily ECM induction. This is supported by the fact that IGFBP-5 has a transactivation domain that facilitates its regulation of transcription [11].

Since the ECM binding domain of IGFBP-5 overlaps with the NLS [42], mutating the NLS also eliminated IGFBP-5 binding to the ECM. Interestingly, even though IGFBP-5 was not able to bind ECM, its induction of fibronectin and collagen deposition in the ECM was similar to that of wild-type IGFBP-5. It is thus likely that the ECM binding domain of IGFBP-5 does not regulate its pro-fibrotic effects, similarly to what has been reported for IGFBP-3 [28]. Mutation of the ECM binding domain of IGFBP-5 reduced smooth-muscle cell response to IGF [42]. Our group recently showed that DOK5/IRS6 is a downstream mediator of IGFBP-5 function [41]. DOK5 expression is increased by IGFBP-5, and DOK5 mediated the pro-fibrotic effects of IGFBP-5. DOK5 is a membrane-associated adaptor protein that likely interacts with a membrane receptor or cytoplasmic protein(s). It is plausible that it is this interaction with an adaptor protein, rather than with the ECM, that is critical for the pro-fibrotic activity of IGFBP-5. It is noteworthy that IGFBP-5 mediated remodeling of the ECM in the involuting mammary gland occurs via interaction with the plasminogen system [43] and is likely tissue-specific.

IGFBP-5 initially was identified as an IGF binding protein, however several functions of IGFBP-5 are IGF-independent. Several groups have reported IGF-independent effects of IGFBP-5 in different cells [11, 17–21, 44] and tissues [45]. We now add promotion of fibrosis as yet another IGF-independent activity of IGFBP-5, validating previous *in vitro* findings using neutralizing anti-IGF-I antibody [9].

## Conclusion

In summary, our findings demonstrate that IGFBP-5 does not require an intact NLS nor high affinity binding to IGF-I to induce a pro-fibrotic phenotype. Further, IGFBP-5 localization to the nucleus is facilitated by its interaction with nucleolin as loss of nucleolin abrogated IGFBP-5 translocation to the nucleus but did not affect is fibrotic activity, further confirming that IGFBP-5 nuclear compartmentalization is not necessary for promoting fibrosis.

## References

[1] Feghali CA, Wright TM. Identification of multiple, differentially expressed messenger RNAs in dermal fibroblasts from patients with systemic sclerosis. Arthritis Rheum. 1999;42(7):1451–7. 10.1002/1529-0131(199907)42:7<1451::AID-ANR19>3.0.CO;2-6 . 10403273

[2] Pilewski JM, Liu L, Henry AC, Knauer AV, Feghali-Bostwick CA. Insulin-like growth factor binding proteins 3 and 5 are overexpressed in idiopathic pulmonary fibrosis and contribute to extracellular matrix deposition. The American journal of pathology. 2005;166(2):399–407. 10.1016/S0002-9440(10)62263-8 15681824 PMC1602317

[3] Yasuoka H, Larregina AT, Yamaguchi Y, Feghali-Bostwick CA. Human skin culture as an ex vivo model for assessing the fibrotic effects of insulin-like growth factor binding proteins. Open Rheumatol J. 2008;2:17–22. 10.2174/1874312900802010017 19088866 PMC2577950

[4] Yasuoka H, Jukic DM, Zhou Z, Choi AM, Feghali-Bostwick CA. Insulin-like growth factor binding protein 5 induces skin fibrosis: A novel murine model for dermal fibrosis. Arthritis Rheum. 2006;54(9):3001–10. 10.1002/art.22084 . 16947625

[5] Yasuoka H, Zhou Z, Pilewski JM, Oury TD, Choi AM, Feghali-Bostwick CA. Insulin-like growth factor-binding protein-5 induces pulmonary fibrosis and triggers mononuclear cellular infiltration. The American journal of pathology. 2006;169(5):1633–42. 10.2353/ajpath.2006.060501 17071587 PMC1780193

[6] Yasuoka H, Yamaguchi Y, Feghali-Bostwick CA. The pro-fibrotic factor IGFBP-5 induces lung fibroblast and mononuclear cell migration. American journal of respiratory cell and molecular biology. 2009;41(2):179–88. 10.1165/rcmb.2008-0211OC 19131643 PMC2715907

[7] Schedlich LJ, Young TF, Firth SM, Baxter RC. Insulin-like growth factor-binding protein (IGFBP)-3 and IGFBP-5 share a common nuclear transport pathway in T47D human breast carcinoma cells. The Journal of biological chemistry. 1998;273(29):18347–52. . 9660801 10.1074/jbc.273.29.18347

[8] Schedlich LJ, Le Page SL, Firth SM, Briggs LJ, Jans DA, Baxter RC. Nuclear import of insulin-like growth factor-binding protein-3 and -5 is mediated by the importin beta subunit. The Journal of biological chemistry. 2000;275(31):23462–70. 10.1074/jbc.M002208200 . 10811646

[9] Yasuoka H, Hsu E, Ruiz XD, Steinman RA, Choi AM, Feghali-Bostwick CA. The fibrotic phenotype induced by IGFBP-5 is regulated by MAPK activation and egr-1-dependent and-independent mechanisms. The American journal of pathology. 2009;175(2):605–15. 10.2353/ajpath.2009.080991 19628764 PMC2716960

[10] Amaar YG, Thompson GR, Linkhart TA, Chen ST, Baylink DJ, Mohan S. Insulin-like growth factor-binding protein 5 (IGFBP-5) interacts with a four and a half LIM protein 2 (FHL2). The Journal of biological chemistry. 2002;277(14):12053–60. 10.1074/jbc.M110872200 . 11821401

[11] Xu Q, Li S, Zhao Y, Maures TJ, Yin P, Duan C. Evidence that IGF binding protein-5 functions as a ligand-independent transcriptional regulator in vascular smooth muscle cells. Circulation research. 2004;94(5):E46–54. 10.1161/01.RES.0000124761.62846.DF . 15001525

[12] Akkiprik M, Hu L, Sahin A, Hao X, Zhang W. The subcellular localization of IGFBP5 affects its cell growth and migration functions in breast cancer. BMC cancer. 2009;9:103 10.1186/1471-2407-9-103 19341485 PMC2670316

[13] Yamaguchi Y, Yasuoka H, Stolz DB, Feghali-Bostwick CA. Decreased caveolin-1 levels contribute to fibrosis and deposition of extracellular IGFBP-5. Journal of cellular and molecular medicine. 2011;15(4):957–69. 10.1111/j.1582-4934.2010.01063.x 20345844 PMC2995014

[14] Overgaard MT, Boldt HB, Laursen LS, Sottrup-Jensen L, Conover CA, Oxvig C. Pregnancy-associated plasma protein-A2 (PAPP-A2), a novel insulin-like growth factor-binding protein-5 proteinase. The Journal of biological chemistry. 2001;276(24):21849–53. 10.1074/jbc.M102191200 . 11264294

[15] Yan X, Baxter RC, Firth SM. Involvement of pregnancy-associated plasma protein-A2 in insulin-like growth factor (IGF) binding protein-5 proteolysis during pregnancy: a potential mechanism for increasing IGF bioavailability. The Journal of clinical endocrinology and metabolism. 2010;95(3):1412–20. 10.1210/jc.2009-2277 . 20103653

[16] Amaar YG, Baylink DJ, Mohan S. Ras-association domain family 1 protein, RASSF1C, is an IGFBP-5 binding partner and a potential regulator of osteoblast cell proliferation. Journal of bone and mineral research: the official journal of the American Society for Bone and Mineral Research. 2005;20(8):1430–9. 10.1359/JBMR.050311 16007340 PMC2897826

[17] Seurin D, Lombet A, Babajko S, Godeau F, Ricort JM. Insulin-like growth factor binding proteins increase intracellular calcium levels in two different cell lines. PloS one. 2013;8(3):e59323 10.1371/journal.pone.0059323 23527161 PMC3602172

[18] Hermani A, Shukla A, Medunjanin S, Werner H, Mayer D. Insulin-like growth factor binding protein-4 and -5 modulate ligand-dependent estrogen receptor-alpha activation in breast cancer cells in an IGF-independent manner. Cellular signalling. 2013;25(6):1395–402. 10.1016/j.cellsig.2013.02.018 . 23499909

[19] Tripathi G, Salih DA, Drozd AC, Cosgrove RA, Cobb LJ, Pell JM. IGF-independent effects of insulin-like growth factor binding protein-5 (Igfbp5) in vivo. FASEB journal: official publication of the Federation of American Societies for Experimental Biology. 2009;23(8):2616–26. 10.1096/fj.08-114124 . 19332648

[20] Cobb LJ, Salih DA, Gonzalez I, Tripathi G, Carter EJ, Lovett F, et al Partitioning of IGFBP-5 actions in myogenesis: IGF-independent anti-apoptotic function. Journal of cell science. 2004;117(Pt 9):1737–46. 10.1242/jcs.01028 . 15075235

[21] Sureshbabu A, Okajima H, Yamanaka D, Tonner E, Shastri S, Maycock J, et al IGFBP5 induces cell adhesion, increases cell survival and inhibits cell migration in MCF-7 human breast cancer cells. Journal of cell science. 2012;125(Pt 7):1693–705. 10.1242/jcs.092882 . 22328518

[22] Imai Y, Moralez A, Andag U, Clarke JB, Busby WH Jr., Clemmons DR. Substitutions for hydrophobic amino acids in the N-terminal domains of IGFBP-3 and -5 markedly reduce IGF-I binding and alter their biologic actions. The Journal of biological chemistry. 2000;275(24):18188–94. 10.1074/jbc.M000070200 . 10766744

[23] Andrews NC, Faller DV. A rapid micropreparation technique for extraction of DNA-binding proteins from limiting numbers of mammalian cells. Nucleic acids research. 1991;19(9):2499 2041787 10.1093/nar/19.9.2499 PMC329467

[24] Santos AM, Jung J, Aziz N, Kissil JL, Pure E. Targeting fibroblast activation protein inhibits tumor stromagenesis and growth in mice. The Journal of clinical investigation. 2009;119(12):3613–25. 10.1172/JCI38988 19920354 PMC2786791

[25] Nam TJ, Busby WH Jr., Rees C, Clemmons DR. Thrombospondin and osteopontin bind to insulin-like growth factor (IGF)-binding protein-5 leading to an alteration in IGF-I-stimulated cell growth. Endocrinology. 2000;141(3):1100–6. 10.1210/endo.141.3.7386 . 10698186

[26] Moralez AM, Maile LA, Clarke J, Busby WH Jr., Clemmons DR. Insulin-like growth factor binding protein-5 (IGFBP-5) interacts with thrombospondin-1 to induce negative regulatory effects on IGF-I actions. J Cell Physiol. 2005;203(2):328–34. 10.1002/jcp.20343 . 15700281

[27] Nam T, Moralez A, Clemmons D. Vitronectin binding to IGF binding protein-5 (IGFBP-5) alters IGFBP-5 modulation of IGF-I actions. Endocrinology. 2002;143(1):30–6. 10.1210/endo.143.1.8596 . 11751588

[28] Jones JI, Gockerman A, Busby WH Jr., Camacho-Hubner C, Clemmons DR. Extracellular matrix contains insulin-like growth factor binding protein-5: potentiation of the effects of IGF-I. J Cell Biol. 1993;121(3):679–87. 7683690 10.1083/jcb.121.3.679 PMC2119570

[29] Ginisty H, Amalric F, Bouvet P. Nucleolin functions in the first step of ribosomal RNA processing. The EMBO journal. 1998;17(5):1476–86. 10.1093/emboj/17.5.1476 9482744 PMC1170495

[30] Bourbon H, Bugler B, Caizergues-Ferrer M, Amalric F. Role of phosphorylation on the maturation pathways of a 100 kDa nucleolar protein. FEBS letters. 1983;155(2):218–22. . 6852233 10.1016/0014-5793(82)80606-6

[31] Lapeyre B, Bourbon H, Amalric F. Nucleolin, the major nucleolar protein of growing eukaryotic cells: an unusual protein structure revealed by the nucleotide sequence. Proceedings of the National Academy of Sciences of the United States of America. 1987;84(6):1472–6. 3470736 10.1073/pnas.84.6.1472 PMC304456

[32] Lischwe MA, Roberts KD, Yeoman LC, Busch H. Nucleolar specific acidic phosphoprotein C23 is highly methylated. The Journal of biological chemistry. 1982;257(24):14600–2. . 7174653

[33] Lischwe MA, Cook RG, Ahn YS, Yeoman LC, Busch H. Clustering of glycine and NG,NG-dimethylarginine in nucleolar protein C23. Biochemistry. 1985;24(22):6025–8. . 4084504 10.1021/bi00343a001

[34] Seinsoth S, Uhlmann-Schiffler H, Stahl H. Bidirectional DNA unwinding by a ternary complex of T antigen, nucleolin and topoisomerase I. EMBO reports. 2003;4(3):263–8. 10.1038/sj.embor.embor770 12634843 PMC1315898

[35] Ma N, Matsunaga S, Takata H, Ono-Maniwa R, Uchiyama S, Fukui K. Nucleolin functions in nucleolus formation and chromosome congression. Journal of cell science. 2007;120(Pt 12):2091–105. 10.1242/jcs.008771 . 17535846

[36] Chen CM, Chiang SY, Yeh NH. Increased stability of nucleolin in proliferating cells by inhibition of its self-cleaving activity. The Journal of biological chemistry. 1991;266(12):7754–8. . 2019600

[37] Hovanessian AG. Midkine, a cytokine that inhibits HIV infection by binding to the cell surface expressed nucleolin. Cell research. 2006;16(2):174–81. 10.1038/sj.cr.7310024 . 16474431

[38] Said EA, Courty J, Svab J, Delbe J, Krust B, Hovanessian AG. Pleiotrophin inhibits HIV infection by binding the cell surface-expressed nucleolin. The FEBS journal. 2005;272(18):4646–59. 10.1111/j.1742-4658.2005.04870.x . 16156786

[39] Shi H, Huang Y, Zhou H, Song X, Yuan S, Fu Y, et al Nucleolin is a receptor that mediates antiangiogenic and antitumor activity of endostatin. Blood. 2007;110(8):2899–906. 10.1182/blood-2007-01-064428 . 17615292

[40] Barel M, Meibom K, Charbit A. Nucleolin, a shuttle protein promoting infection of human monocytes by Francisella tularensis. PloS one. 2010;5(12):e14193 10.1371/journal.pone.0014193 21152024 PMC2995743

[41] Yasuoka H, Yamaguchi Y, Feghali-Bostwick CA. The membrane-associated adaptor protein DOK5 is upregulated in systemic sclerosis and associated with IGFBP-5-induced fibrosis. PloS one. 2014;9(2):e87754 10.1371/journal.pone.0087754 24551065 PMC3923757

[42] Parker A, Rees C, Clarke J, Busby WH Jr., Clemmons DR. Binding of insulin-like growth factor (IGF)-binding protein-5 to smooth-muscle cell extracellular matrix is a major determinant of the cellular response to IGF-I. Mol Biol Cell. 1998;9(9):2383–92. 9725901 10.1091/mbc.9.9.2383 PMC25505

[43] Tonner E, Allan G, Shkreta L, Webster J, Whitelaw CB, Flint DJ. Insulin-like growth factor binding protein-5 (IGFBP-5) potentially regulates programmed cell death and plasminogen activation in the mammary gland. Advances in experimental medicine and biology. 2000;480:45–53. . 10959408 10.1007/0-306-46832-8_5

[44] Perks CM, McCaig C, Holly JM. Differential insulin-like growth factor (IGF)-independent interactions of IGF binding protein-3 and IGF binding protein-5 on apoptosis in human breast cancer cells. Involvement of the mitochondria. Journal of cellular biochemistry. 2000;80(2):248–58. . 11074596 10.1002/1097-4644(20010201)80:2<248::aid-jcb140>3.0.co;2-4

[45] Flint DJ, Tonner E, Allan GJ. Insulin-like growth factor binding proteins: IGF-dependent and-independent effects in the mammary gland. Journal of mammary gland biology and neoplasia. 2000;5(1):65–73. . 10791769 10.1023/a:1009567316520

